# A novel approach for a joint analysis of isomiR and mRNA expression data reveals features of isomiR targeting in breast cancer

**DOI:** 10.3389/fgene.2022.1070528

**Published:** 2022-12-01

**Authors:** Stepan Nersisyan, Anton Zhiyanov, Narek Engibaryan, Diana Maltseva, Alexander Tonevitsky

**Affiliations:** ^1^ Faculty of Biology and Biotechnology, HSE University, Moscow, Russia; ^2^ Shemyakin-Ovchinnikov Institute of Bioorganic Chemistry, Russian Academy of Sciences, Moscow, Russia; ^3^ Art Photonics GmbH, Berlin, Germany

**Keywords:** isomiR, miRNA, targeting, breast cancer, TCGA

## Abstract

A widely used procedure for selecting significant miRNA-mRNA or isomiR-mRNA pairs out of predicted interactions involves calculating the correlation between expression levels of miRNAs/isomiRs and mRNAs in a series of samples. In this manuscript, we aimed to assess the validity of this procedure by comparing isomiR-mRNA correlation profiles in sets of sequence-based predicted target mRNAs and non-target mRNAs (negative controls). Target prediction was carried out using RNA22 and TargetScan algorithms. Spearman’s correlation analysis was conducted using miRNA and mRNA sequencing data of The Cancer Genome Atlas Breast Invasive Carcinoma (TCGA-BRCA) project. Luminal A, luminal B, basal-like breast cancer subtypes, and adjacent normal tissue samples were analyzed separately. Using the sets of putative targets and non-targets, we introduced adjusted isomiR targeting activity (ITA)—the number of negatively correlated potential isomiR targets adjusted by the background (estimated using non-target mRNAs). We found that for most isomiRs a significant negative correlation between isomiR-mRNA expression levels appeared more often in a set of predicted targets compared to the non-targets. This trend was detected for both classical seed region binding types (8mer, 7mer-m8, 7mer-A1, 6mer) predicted by TargetScan and the non-classical ones (G:U wobbles and up to one mismatch or unpaired nucleotide within seed sequence) predicted by RNA22. Adjusted ITA distributions were similar for target sites located in 3′-UTRs and coding mRNA sequences, while 5′-UTRs had much lower scores. Finally, we observed strong cancer subtype-specific patterns of isomiR activity, highlighting the differences between breast cancer molecular subtypes and normal tissues. Surprisingly, our target prediction- and correlation-based estimates of isomiR activities were practically non-correlated with the average isomiR expression levels neither in cancerous nor in normal samples.

## Introduction

MiRNAs are short non-coding RNAs that post-transcriptionally regulate gene expression. A mature miRNA binds a target mRNA, which results in mRNA degradation or translational repression (Bartel, 2009). Target recognition by a miRNA heavily depends on nucleotides 2–7 at the 5′-end of the miRNA called seed region. Specifically, high complementarity of seed region with target sequence is required for successful miRNA-mRNA binding (Bartel, 2009). Gene expression regulation by miRNA molecules plays a vital role in cancer development, progression, and metastasis (Di Leva et al., 2014; Reddy, 2015).

The application of small RNA sequencing technology led researchers to identify miRNA isoforms (isomiRs): variants of a mature miRNA differing from each other by a few nucleotides at 5′, 3′, or both ends (Tomasello et al., 2021). While the complete pathway of isomiR biogenesis has not yet been discovered, several mechanisms have been shown to contribute to the generation of isomiRs. These mechanisms include but are not limited to heterogeneous cleavage of pri- and pre-miRNA hairpins by Drosha and Dicer enzymes, respectively, post-transcriptional nucleotide addition to the 3′-ends of miRNAs by nucleotidyl transferases (Tomasello et al., 2021). Importantly, length variation at a 5′-end of an isomiR alters the isomiR’s targetome since the seed sequence is modified. Experimental validation of non-canonical 5′-isomiR targets was previously conducted for miR-9 (Tan et al., 2014), miR-34/449 (Mercey et al., 2017), miR-101 (Llorens et al., 2013), miR-183 (Telonis et al., 2015), and miR-411 (van der Kwast et al., 2020).

A common strategy for the bioinformatics analysis of the miRNA/isomiR targeting involves a two-step procedure. First, target mRNAs for a given isomiR are predicted based on nucleotide sequences. Several broadly used tools are available for making such predictions, including RNA22 (Miranda et al., 2006), TargetScan (McGeary et al., 2019), miRDB (Chen and Wang, 2020), DIANA-microT (Reczko et al., 2012) and others (Riolo et al., 2020). Then, isomiR and target (mRNA- or protein-level) expression profiles in a set of samples are used to select putative interactions supported by a significant negative correlation. Such a strategy was previously used, e.g., to discover critical miRNA-mRNA interactions in breast cancer (Telonis and Rigoutsos, 2018; Shkurnikov et al., 2019; Nersisyan et al., 2021b), colorectal cancer (Nersisyan et al., 2021a), prostate cancer (Magee et al., 2018) and chronic lymphocytic leukemia (Cimmino et al., 2005).

However, to the best of our knowledge, there were no attempts at unbiased evaluation of this strategy’s validity. Muniategui with coauthors compared different expression correlation-based miRNA target prediction methods using databases of experimentally validated miRNA targets (Muniategui et al., 2013). While this approach allowed the authors to estimate the sensitivity of the target prediction, the validated miRNA target databases are significantly biased towards well-studied miRNAs and highly complementary interactions in the case of low-throughput experiments (e.g., luciferase reporter assays). While CLIP-seq datasets could serve as an unbiased source of validated interactions, there is a problem related to the absence of gene expression repression for a considerable portion of identified interactions (Chu et al., 2020).

The main objective of the present study was to assess the validity of performing isomiR-mRNA or miRNA-mRNA correlation analysis on the results of sequence-based target prediction. For that, we comprehensively compared miRNA-mRNA expression level correlations between predicted targets and negative controls (briefly, mRNAs that do not contain miRNA seed region binding sites). The analysis was conducted using miRNA and mRNA sequencing data of primary breast cancer (BC) samples and adjacent normal tissues available in The Cancer Genome Atlas Breast Invasive Carcinoma (TCGA-BRCA) project (PAM50 molecular subtypes of BC were analyzed separately). We aimed to perform unbiased and data-driven analysis, so we predicted isomiR targets with two bioinformatics tools based on different ideas, with both classical and non-classical seed region binding types. We also considered targeting within mRNA coding sequences (CDS) and 5′-UTRs.

## Materials and methods

### TCGA data acquisition and processing

Count-level TCGA isomiR expression data ( n=11089 samples) were downloaded from the IsoMiRmap tool (Loher et al., 2021) official website (https://cm.jefferson.edu/isomirmap/). Reads exclusively mapped to the miRNA space were selected for further processing. Transcript-level mRNA-seq data for n=10530 TCGA samples were downloaded from the UCSC Xena portal (Goldman et al., 2020) (https://xenabrowser.net/), TOIL RSEM expected_count dataset built on GENCODE 23 human reference genome was used.

Normalization of both miRNA-seq and mRNA-seq TCGA count-level data was performed using the median of ratios algorithm implemented as the “estimateSizeFactors” function in the DESeq2 R package (Love et al., 2014); “fpm” and “fpkm” functions were used to generate the final normalized expression tables for miRNA-seq and mRNA-seq data, respectively (i.e., mRNA-seq data was additionally normalized by the transcript lengths). Finally, we applied $\log_{2}\left(x+1\right)$ transformation for both miRNA-seq and mRNA-seq data.

PAM50 molecular subtyping results of primary breast cancer samples and normal tissues annotation were extracted from the UCSC Xena portal. We set a minimum of 100 sample thresholds for all subtypes to eliminate possible sample size-related biases in the downstream analysis. As a result, four sample groups were considered: luminal A primary tumors ( n=561 samples), luminal B primary tumors ( n=210 ), basal-like primary tumors ( n=183 ), and adjacent normal tissues ( n=104 ). Sample identifiers and corresponding group labels are available in Supplementary Table S1.

Since 3′-end isomiR variations do not alter the isomiR targetome much (seed region not affected), we summed up the expression of isomiRs originating from the same miRNA, which have identical 5′-end sequences (5′-isomiRs). We used our previous notation for 5′-isomiRs (Nersisyan et al., 2022b): a number after the “|” symbol stands for a shift from the canonical 5′-end in the 5′–3′ direction. For example, hsa-miR-192-5p|+1 differs from the canonical hsa-miR-192-5p miRNA by the absence of the first nucleotide on its 5′-end.

For further processing, we selected 139 5′-isomiRs with the highest median expression—this was a minimum number of isomiRs that covered 99% of the total median isomiR expression. The used threshold corresponded to approximately 100 DESeq2-normalized FPM. A less strict thresholding was used for the mRNA-seq data: transcripts with zero reads in more than half of the analyzed samples were discarded. The final list of analyzed 5′-isomiRs and their median expression levels is available in Supplementary Table S2.

### IsomiR target prediction

Two tools were used to predict 5′-isomiR targets: RNA22 (Miranda et al., 2006; Loher and Rigoutsos, 2012) and TargetScan 7.2 (Agarwal et al., 2015). Precomputed RNA22 predictions for the canonical miRNAs were downloaded from the official tool’s website (https://cm.jefferson.edu/rna22-full-sets-of-predictions/; ENSEMBL 96 and miRBase 22 version). For the remaining 30 non-canonical isomiRs, a program that allows one to submit target prediction batch requests was used (https://cm.jefferson.edu/rna22/Interactive/remoteRNA22v2.zip, ENSEMBL 96 transcript sequences). The default target predictions settings were used. Since human reference transcriptome versions differed for mRNA-seq reads mapping and isomiR target prediction, we selected transcripts with identical sequences in both versions for further analysis.

The set of predicted isomiR-mRNA interactions was classified into five types based on seed region binding motifs: 8mer, 7mer-m8, 7mer-A1, 6mer (classical seed binding types), and other (including unlimited G:U wobbles and up to one mismatch or unpaired nucleotide in a seed region). Custom TargetScan predictions were done as previously described (Nersisyan et al., 2022a): publicly available Perl scripts and 3′-UTR sequences were downloaded from the official website (https://www.targetscan.org/cgi-bin/targetscan/data_download.vert72.cgi). Assessment of binding with imperfect seed complementarity (3′-compensatory sites) was not possible with TargetScan custom prediction mode. The results of RNA22 and TargetScan predictions are available in figshare repository (https://doi.org/10.6084/m9.figshare.21579729.v1). MiRTarBase 9 database (Huang et al., 2022) was used to retrieve experimentally verified miRNA-mRNA interactions (luciferase reporter assays).

### Composition of isomiR non-target mRNA sets

For a given 5′-isomiR, we composed a set of all mRNAs which do not contain the 6mer seed region binding site in mRNA sequences (including an unlimited number of G:U wobbles). The computational approach for solving this problem was based on the composition of the hash table, which mapped all possible 6mers to their positions in all mRNA sequences. The set of negative control interactions is available in Supplementary Data 1.

### Calculating isomiR targeting activity (ITA) and adjusted ITA

We calculated Spearman’s correlation coefficients and corresponding p -values between all 5′-isomiR and mRNA expression levels (separately for the four groups of BC samples). Benjamini–Hochberg procedure was used to adjust p -values.

For a given 5′-isomiR, we denote the number of predicted targets as $n_{tar}$ and the number of predicted targets supported by a significant negative correlation of expression levels ( r<−0.3, FDR<0.05 ) as $m_{tar}$ ( $m_{tar}\leq n_{tar}$ ). The analogous terms were used for the isomiR non-targets: $n_{nontar}$ (number of non-targets predicted) and $m_{nontar}$ (number of anti-correlated non-targets: r<−0.3, FDR<0.05 ). For the downstream text, we denote $m_{tar}$ as *isomiR targeting activity* (*ITA*) and $m_{tar}-n_{tar}\frac{m_{nontar}}{n_{nontar}}$ as *adjusted ITA*. The statistical significance of an isomiR activity was assessed with the one-sided Fisher’s exact test applied to the following contingency table: $\left(\begin{array}{cc} m_{tar} & n_{tar}-m_{tar}\\ m_{nontar} & n_{nontar}-m_{nontar} \end{array}\right)$

Benjamini–Hochberg procedure was used to adjust p -values.

### Differential expression analysis

We used DESeq2 1.36 (Love et al., 2014) to identify differentially expressed isomiRs for each pair of the considered sample groups (luminal A, luminal B, and basal-like BCs, normal samples). An isomiR was considered as differentially expressed if the fold change was lower than 0.5 or higher than two and the adjusted p -value was lower than 0.05.

### Statistical analysis and programming

We used SciPy stats 1.9 (Virtanen et al., 2020) for conducting statistical analysis (Spearman’s correlation, Fisher’s exact test). Pandas 1.3 (McKinney, 2010) and NumPy 1.23 (Harris et al., 2020) were used for miscellaneous computations. Plots were constructed with Seaborn 0.11 (Waskom, 2021) and ggVennDiagram (Gao et al., 2021).

## Results

### Bioinformatics analysis workflow

**FIGURE 1:**
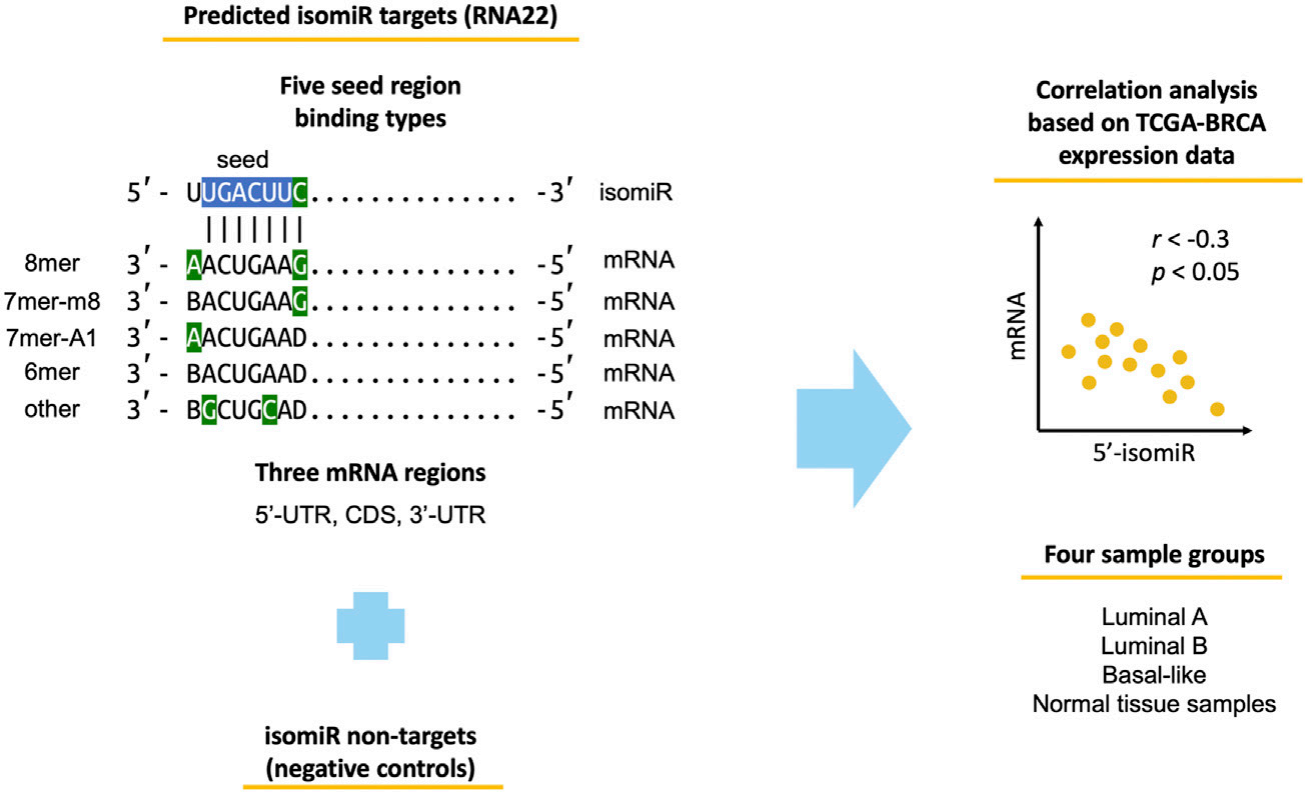
Workflow of the conducted analysis. Blue-colored nucleotides stand for the classical miRNA seed region. Green-colored nucleotides stand for seed-type-specific features (complementarity of nucleotide 8 for 8mer and 7mer-m8, adenine in position one for 8mer and 7mer-A1, G:U wobble and C:U mismatch for other). B stands for any nucleotide except adenine, D—any nucleotide except cytosine.

The study workflow is outlined in Figure 1. We used miRNA-seq and mRNA-seq data of TCGA-BRCA primary tumor samples corresponding to three molecular subtypes (luminal A, luminal B, basal-like) and adjacent normal tissue samples. We identified 139 highly expressed 5′-isomiRs and predicted their target mRNAs using two sequence-based target prediction tools: RNA22 and TargetScan. The tools are based on different ideas. Namely, TargetScan favors evolutionary conserved binding sites, relies on the classical seed region binding types (8mer, 7mer-m8, 7mer-A1, 6mer), and predicts targets only within 3′-UTR. In contrast, RNA22 is a pattern-based method, which does not consider target site conservation, predicts targets within 5′-UTR, CDS, and 3′-UTR, and is significantly more tolerant to mismatches in a seed region. Aside from target predictions, we also composed sets of non-targets (i.e., negative controls) for each 5′-isomiR. Non-targets were defined as mRNAs that do not contain regions complementary to an isomiR’s seed region (including possible G:U wobbles). Finally, we calculated Spearman’s correlation coefficient between expression levels of all 5′-isomiRs and their targets and non-targets.

### Differences between the sets of RNA22-and TargetScan-predicted targets

**FIGURE 2:**
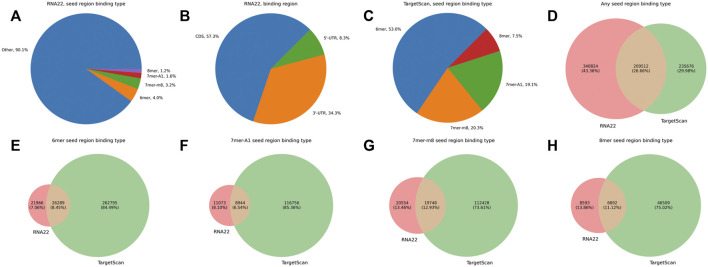
Comparison of RNA22-and TargetScan-predicted isomiR-mRNA interactions. **(A)** percentages of different seed region binding types in RNA22-predicted interaction sites. **(B)** percentages of different mRNA regions in RNA22-predicted interaction sites. **(C)** percentages of different seed region binding types in TargetScan-predicted interaction sites. **(D)** the mutual arrangement between the sets of predicted isomiR-mRNA interactions (not individual interacting sites) by RNA22 and TargetScan. **(E–H)**: the mutual arrangement between the sets of RNA-22 and TargetScan-predicted isomiR-mRNA interactions containing specific seed region binding sites (6mer, 7mer-A1, 7mer-m8, 8mer). For constructing plots **(D–H)**, we considered only these transcripts which were included in the input of both RNA22 and TargetScan.

Before analyzing the joint expression profiles of isomiRs and their putative targets, we compared the sets of predicted isomiR-target interactions between RNA22 and TargetScan tools. Only 9.9% of RNA22-predicted target sites corresponded to the classical seed region binding (4.0% for 6mer, 3.2% for 7mer-m8, 1.6% for 7mer-A1, and 1.2% for 8mer), while the vast majority of putative targets had non-classical seed binding (90.1%, Figure 2A). High numbers of target sites were found in 3′-UTR and CDS (57.3% and 34.3%, respectively), while 5′-UTR binding was reported less frequently (8.3%, Figure 2B). TargetScan predictions included only classical seed region binding types within 3′-UTRs, with a prevalence of 6mer sites (53%, Figure 2C). RNA22 predicted 1.2 times more isomiR-mRNA interactions than TargetScan, and the tools shared 26.7% of the putative interactions (Figure 2D). At the same time, RNA22 missed the majority of classical seed region binding sites in 3′-UTRs compared to TargetScan (Figures 2E–H). For the downstream analysis, we used the union of RNA22-and TargetScan-predicted isomiR-mRNA interactions. With such a target prediction strategy, we were able to cover 89.1% out of 1,608 experimentally validated interactions between canonical miRNAs and their target mRNAs. In particular, 49.6% of validated interactions were predicted by both RNA22 and TargetScan, 6.5%—only by RNA22, and 33.0%—only by TargetScan.

isomiRs tend to stronger anti-correlate with the putative targets rather than with the non-targets.

To systematically compare correlation profiles of 5′-isomiRs with their targets and non-targets, we introduced two measures of isomiR activity. The first one, denoted as *isomiR targeting activity* (*ITA*), was calculated as a number of predicted targets, which were additionally supported by the significant negative correlation. However, a negative correlation between expression levels of a 5′-isomiR and its target gene could be explained by non-random side effects, such as opposite-sided regulation of both molecules by a transcription factor. Therefore, to estimate the number of such false positive interactions (i.e., negatively correlated 5′-isomiRs and their target genes with no direct effect), we used the non-target mRNA sets. Specifically, *background ITA* was calculated as the fraction of non-targets that were significantly anti-correlated with the 5′-isomiR, multiplied by the number of predicted isomiR targets. Finally, *adjusted ITA* was calculated by subtracting background ITA from unadjusted ITA. In other words, adjusted ITA reflects the difference between the number of negatively correlated potential isomiR targets and the expected number of negatively correlated non-targets.

Note that near-zero or negative adjusted ITA does not imply the absence of targeting. For example, consider the synthetic case where the only target of a 5′-isomiR is a hub TF with multiple downstream target genes, which are not direct targets of the isomiR. In this case, unadjusted ITA equals one, while background activity is much greater since the isomiR is anti-correlated with TF targets by transitivity. Thus, the adjusted ITA of the isomiR is negative despite having one functional target.

**FIGURE 3:**
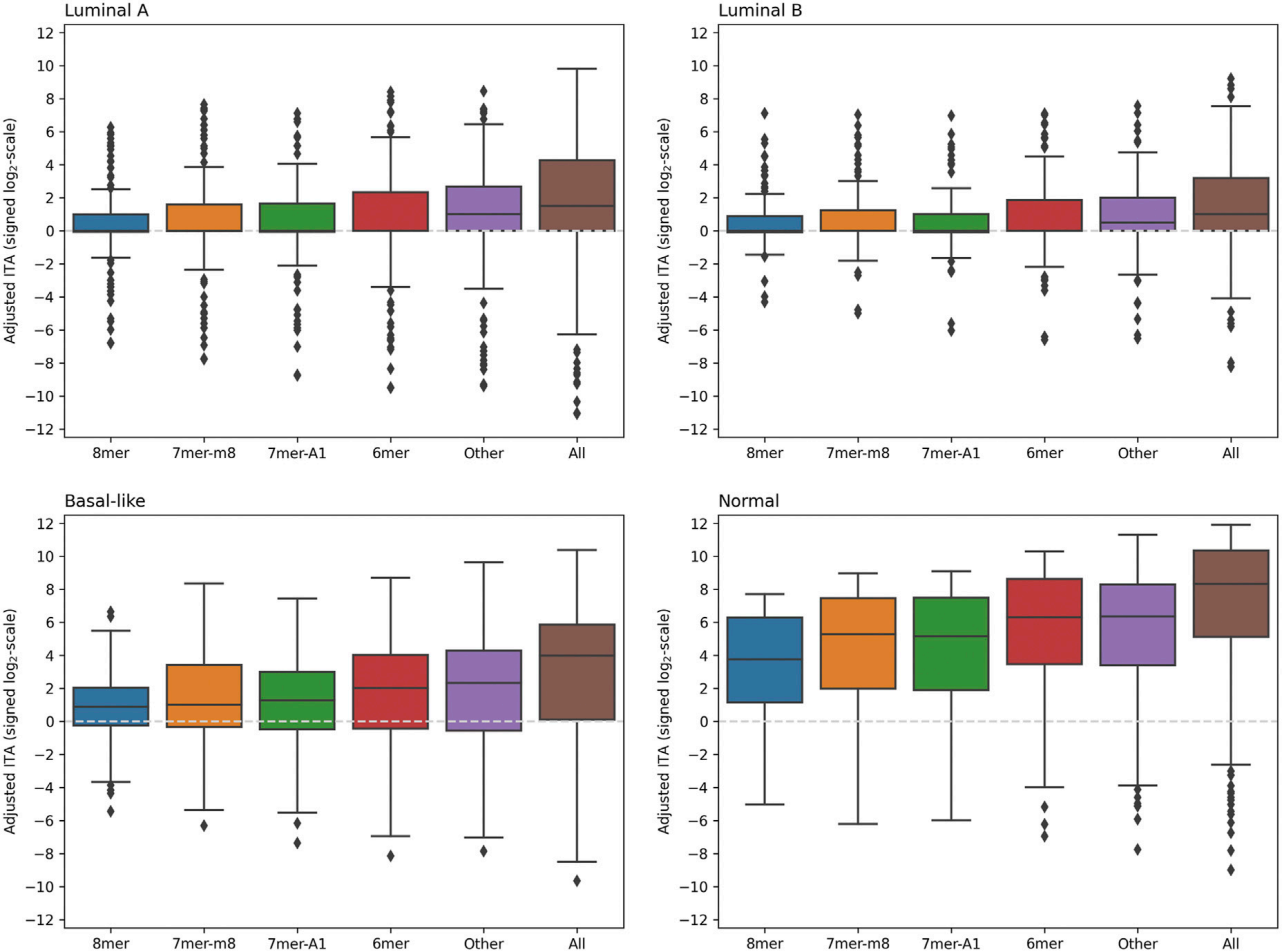
The distribution of adjusted ITA values in four groups of samples. The union of RNA22 and TargetScan predictions was used, including RNA22-predicted target sites with non-classical seed binding (labeled as “Other” on x -axis) and sites in CDS and 5′-UTR. Adjusted ITA values ( y -axis) were calculated for each 5′-isomiR and reflect the number of anti-correlated predicted targets adjusted for the background anti-correlations (background was estimated using non-target transcript sequences). To embed positive and negative adjusted ITA values in the logarithmic scale, we applied the following signed log transformation: $sgn\left(y\right)*\log_{2}\left(\left|y\right|+1\right)$ .

A comparison of adjusted ITA values calculated using 5′-isomiR and mRNA expression levels for the union of RNA22 and TargetScan predictions (including all seed binding types and mRNA regions) is shown in Figure 3. As can be seen, the distributions were essentially skewed toward the positive direction for each seed region binding type and each sample group. Thus, for the majority of 5′-isomiRs, ITA was higher than the background correlations level. Notably, adjusted ITA values were dramatically higher in the normal tissues compared to the cancerous ones. Thus, we observed global downregulation of isomiR targeting in breast cancer. The tables with adjusted ITA scores (union of RNA22 and TargetScan) for three BC subtypes and normal samples are available in Supplementary Table S3-S6.

In consistence with the previous section, RNA22 and TargetScan had different contributions to the correlation analysis. Namely, in the case of RNA22 predictions, non-classical seed binding types (referred to as “Other” in Figure 3) had significantly higher adjusted ITA scores compared to the four classical seed types (Supplementary Figure S1). Moreover, in the case of luminal A and luminal B BC subtypes, the whole distribution of adjusted ITA values (except outliers) was concentrated near zero, which was not the case for the “Other” binding type. Near-equal adjusted ITA scores were found for the 3′-UTR and CDS binding, while adjusted ITA in 5′-UTRs was systematically lower. In contrast to the RNA22 case, an essential mass of the TargetScan-based adjusted ITA distribution was in the positive zone for each classical seed binding type (Supplementary Figure S2). Thus, combining two target prediction algorithms ultimately allowed us to make valid target predictions both for classical and non-classical seed binding sites. It is also worth noting that adjusted ITA scores calculated separately using RNA22 and TargetScan were highly correlated: Spearman’s r=0.70 , $p=5.25\times 10^{-22}$ for Luminal A, r=0.51 , $p=1.73\times 10^{-10}$ for Luminal B, r=0.88 , $p=3.54\times 10^{-47}$ for Basal-like, and r=0.85 , $p=1.31\times 10^{-39}$ for Normal samples.

We were not able to identify any further subgroups within the predicted targets, which would have significantly different adjusted ITA scores. The tested groupings included partitioning of RNA22-predicted interactions by ten quantiles of binding energy, separating RNA22-predicted non-classical target sites by a number of G:U wobbles and mismatches, and partitioning of TargetScan-predicted interactions by ten quantiles of weighted context++ score.

### IsomiR activities markedly vary across BC subtypes and normal tissues

**FIGURE 4:**
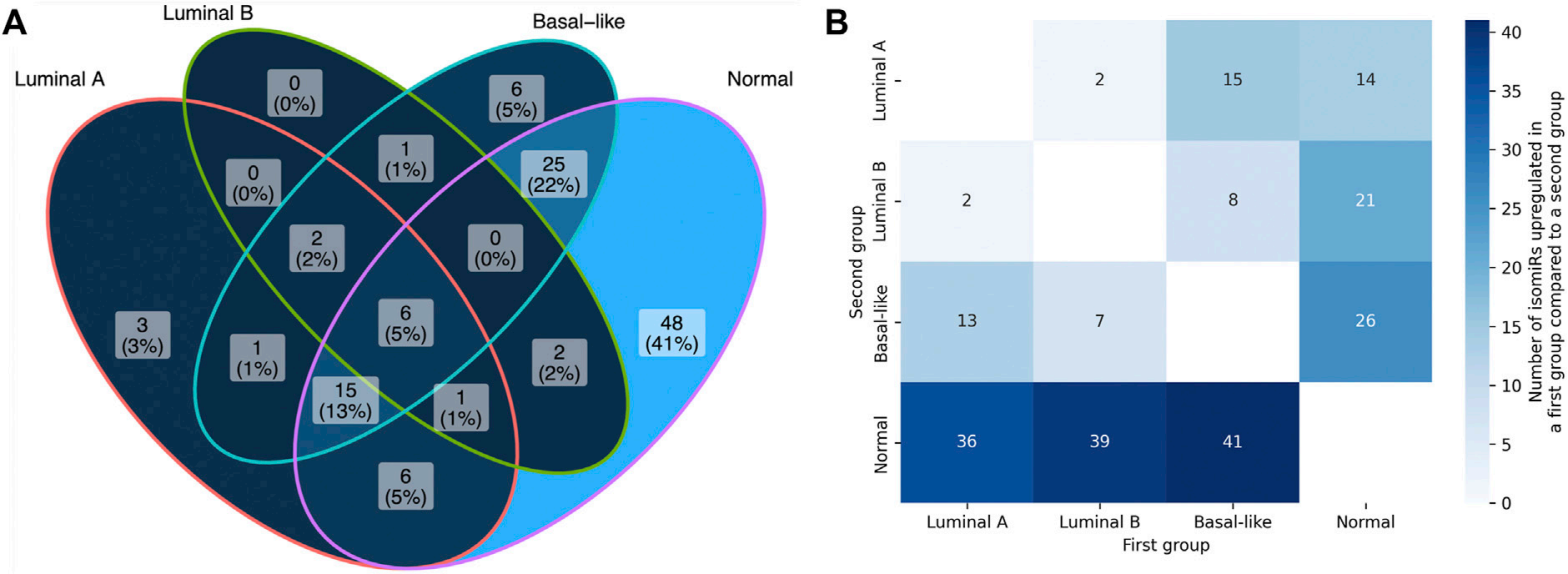
Differential 5′-isomiRs activity and expression in four analyzed sample groups. **(A)** the mutual arrangement of the sets of significantly active 5′-isomiRs (adjusted ITA FDR <0.05) in four sample groups. **(B)** the results of pairwise differential 5′-isomiR expression analysis. Each cell contains a quantitty of differentially expressed isomiRs in a corresponding comparison (fold change >2, FDR <0.05).

Comparison of the numbers of anti-correlated isomiR-mRNA pairs between the sets of predicted targets and non-targets with Fisher’s exact test allowed us to identify 5′-isomiRs with a statistically significant activity over the background. The mutual arrangement of the significant isomiR sets for four sample groups (luminal A, luminal B, basal-like, normal) is presented in Figure 4A. As can be seen, isomiR activity showed clear subtype-specific patterns. Consistently with the previous section, the maximum number of significantly active isomiRs was detected in normal breast tissue samples: 103 out of 139 considered isomiRs. Normal tissues were followed by the basal-like (56 isomiRs), luminal A (34 isomiRs), and luminal B (12 isomiRs) BCs. Only eight isomiRs were significantly active in all BC subtypes: hsa-miR-17-3p|0, hsa-miR-30b-5p|0, hsa-miR-93-5p|0, hsa-miR-101-3p|-1, hsa-miR-106b-5p|0, hsa-miR-182-5p|0, hsa-miR-200b-3p|0, and hsa-miR-210-3p|0.

We then wondered whether the results of differential isomiR activity are in line with expression levels of these isomiRs in four considered sample groups. First, we noticed that the unsupervised clustering of samples based on the isomiR expression profiles was similar enough to the four considered sample groups (importantly, normal samples clustered separately from the cancerous ones, Supplementary Figure S3). Then, we conducted differential isomiR expression analysis for each pair of sample groups (Figure 4B). In contrast to the higher number of active isomiRs in normal tissues, we saw a higher number of isomiR upregulation events in cancer than downregulation events (this held for each BC subtype). The other interesting observation could be made while comparing luminal A and luminal B subtypes: only four isomiRs passed the differential expression significance thresholds (fold change and adjusted p -value), while the Jaccard index for the sets of the active isomiRs (9 isomiRs in the intersection divided by 37 isomiRs in the union) was equal to just 0.24. The detailed analysis of intersections between the sets of active/not active and upregulated/downregulated isomiRs for each pair of sample groups also did not allow us to find any strong associations between an isomiR’s expression level and significance of adjusted ITA score (Supplementary Figure S4). Finally, it is worth noting that we did not find a correlation between adjusted ITA scores and isomiR median expression levels neither in cancerous nor in normal samples (Spearman’s r<0.18 ). Thus, the analyzed data told us that the variation of isomiR expression levels and correlations between isomiRs and putative targets constitute two independent dimensions.

## Discussion

Correlation analysis applied to expression data of miRNAs and mRNAs in a set of samples is widely used to select important miRNA-mRNA interactions. In this manuscript, we assessed the validity of this approach by performing a comprehensive comparison of correlations between the sets of predicted isomiR targets and non-targets. Ultimately, we found that for most isomiRs, the number of anti-correlating mRNAs was higher among predicted targets rather than non-targets. These observations support the validity of the commonly used bioinformatics approach, consisting in searching for negative correlations among sequence-based predicted isomiR-mRNA interactions.

We used two commonly applied sequence-based miRNA target prediction tools to conduct bioinformatics analysis: RNA22 and TargetScan. TargetScan focuses on four classical seed region binding types (8mer, 7mer-m8, 7mer-A1, 6mer) and 3′-UTR targeting. In contrast, RNA22 mainly predicted sites with non-classical seed binding and many sites within mRNA CDS and 5′-UTR. Indeed, four classical sequence types show striking enrichment in large-scale studies involving miRNA overexpression and transcriptome/proteome profiling. Specifically, Lim with co-authors overexpressed miR-1 and miR-124 in HeLa cells and found the most enriched nucleotide motifs in downregulated transcripts detected by microarray analysis—the answer was 6mer seed region for both miRNAs (Lim et al., 2005). Similar results were further derived for five miRNAs using a proteomic approach (Selbach et al., 2008), and for 25 miRNAs using RNA sequencing (Liu and Wang, 2019). Indeed, such experimental setups do not provide evidence of direct miRNA-mRNA interactions. Several Ago-CLIP methods were developed to close this gap, including HITS-CLIP (Chi et al., 2009) and PAR-CLIP (Hafner et al., 2010). Concordantly, motif search in the datasets generated by both methods led researchers to the miRNA seed sequences. However, all the mentioned experiments provided sufficient evidence of efficient targeting despite unpaired nucleotides/bulges in a seed region, and successful targeting in mRNA coding sequences. Specifically, G-bulged sites were present in more than 15% of identified interactions by HITS-CLIP in a mouse brain and were shown to be conserved (Chi et al., 2012); Hafner et al. reported about 7% of non-classical seed sites in PAR-CLIP data (Hafner et al., 2010). Both HITS-CLIP and PAR-CLIP datasets supported extensive miRNA binding within coding sequences of mRNAs. Ribosome profiling data upon miRNA transfection suggested functional differences in CDS and 3′-UTR miRNA binding: CDS sites were more associated with inhibiting translation, while 3′-UTR sites were better at initiating mRNA degradation (Hausser et al., 2013). In a recent study, McGeary et al. used their novel K_d_-based mathematical model to conclude that CDS-located sites have 5.5-folds lower affinity compared to the 3′-UTR sites (McGeary et al., 2019). Target repression through 5′-UTR binding was also reported (Lytle et al., 2007).

Importantly, our analysis was in agreement with both models: the adjusted ITA scores were skewed towards the positive direction in cases of both RNA22-and TargetScan-predicted targets (though 5′-UTR targeting was predicted to be much less efficient compared to 3′-UTR and CDS). However, one limitation of the conducted analysis should be noted. Namely, a single mRNA could contain more than one binding site for a given isomiR, and these sites could be localized in different parts of mRNA or have different seed region binding types. In this case, the same isomiR-mRNA pair was considered in all corresponding designs.

With the use of the developed ITA scores, we were able to select isomiRs that were anti-correlated with a statistically significant number of predicted targets over the background. It turned out that the sets of the most active isomiRs poorly overlapped between the three subtypes of BC and the normal tissue samples. Only eight common 5′-isomiRs for three cancer subtypes were found. Based on these results, we emphasize the importance of considering sample heterogeneity while performing isomiR-mRNA correlation analysis for cancer data. Another striking observation was the absence of correlation between the adjusted ITA scores and the median isomiR expression levels in all considered sample groups. These results could mean that the expression level of a 5′-isomiR is not the main determinant of its functional activity (at least when the set of highly expressed isomiRs is considered). Alternative mechanisms controlling the miRNA targeting were reported. For example, Kim with co-authors recently reported that RNA-binding proteins could significantly enhance miRNA targeting efficiency by making a secondary structure of a target site accessible to the miRNA-Argonaute complex (Kim et al., 2021). Further experiments are warranted to understand the nature of correlation-based isomiR activity in BC samples.

## Data Availability

The original contributions presented in the study are included in the article/Supplementary Material, further inquiries can be directed to the corresponding author.
