# Supplementary material for: A novel approach for a joint analysis of isomiR and mRNA expression data reveals features of isomiR targeting in breast cancer
Source: Front Genet. 2022 Dec 1;13:1070528. doi: 10.3389/fgene.2022.1070528 (PMC9751988; doi:10.3389/fgene.2022.1070528)
Supplement: Supplementary file 2 [file Image1.pdf]

Supplementary Figures

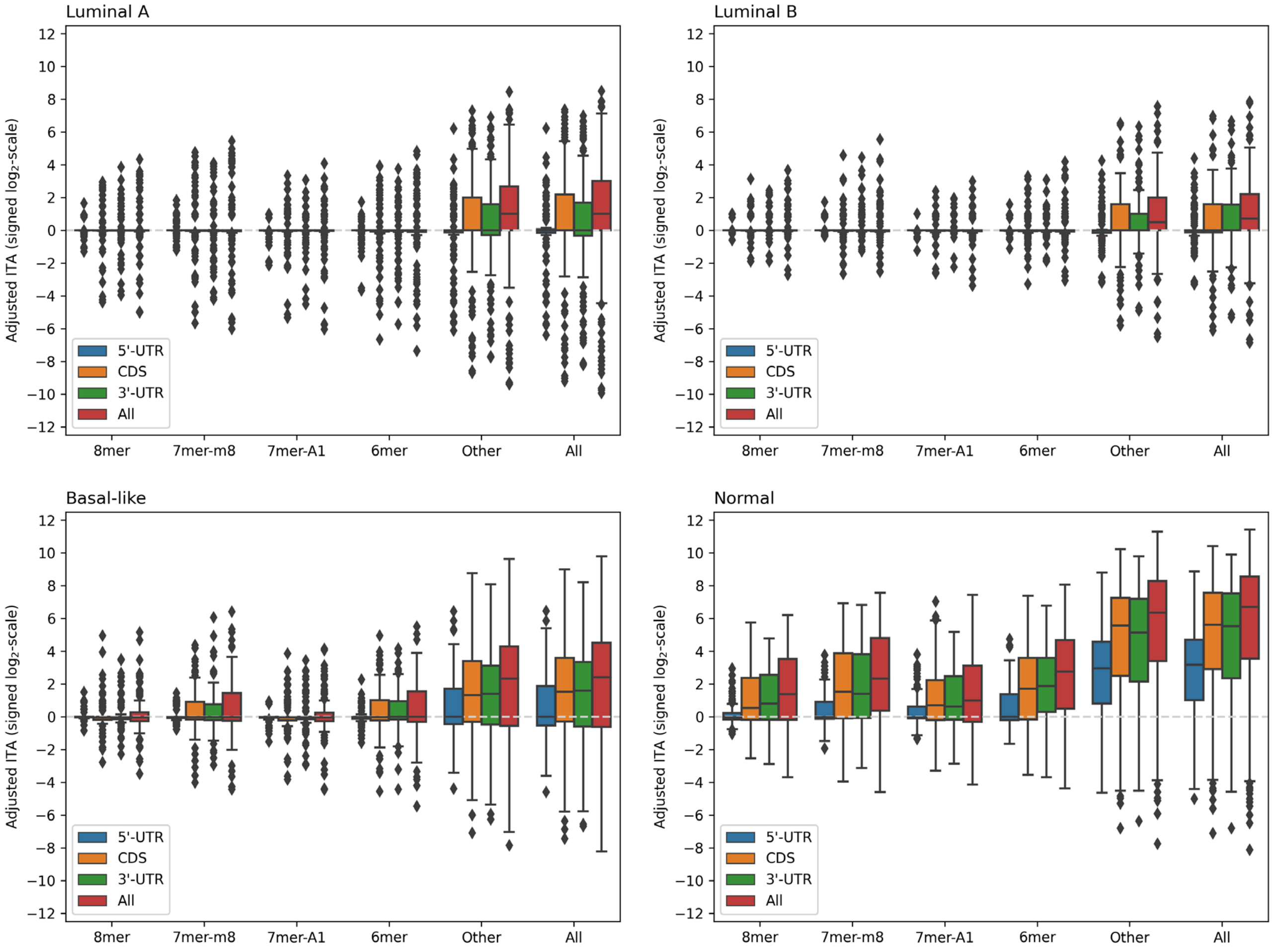

Supplementary Figure 1. The distribution of adjusted ITA values in four groups of samples (only RNA22 predictions). RNA22-predicted target sites with non-classical seed binding were labeled as “Other” on x-axis. Adjusted ITA values (y-axis) were calculated for each 5′-isomiR and reflect the number of anti-correlated predicted targets adjusted for the background anti-correlations (background was estimated using non-target transcript sequences). To embed positive and negative adjusted ITA values in the logarithmic scale, we applied the following signed log transformation: $sgn(y) * log_2(|y| + 1)$.

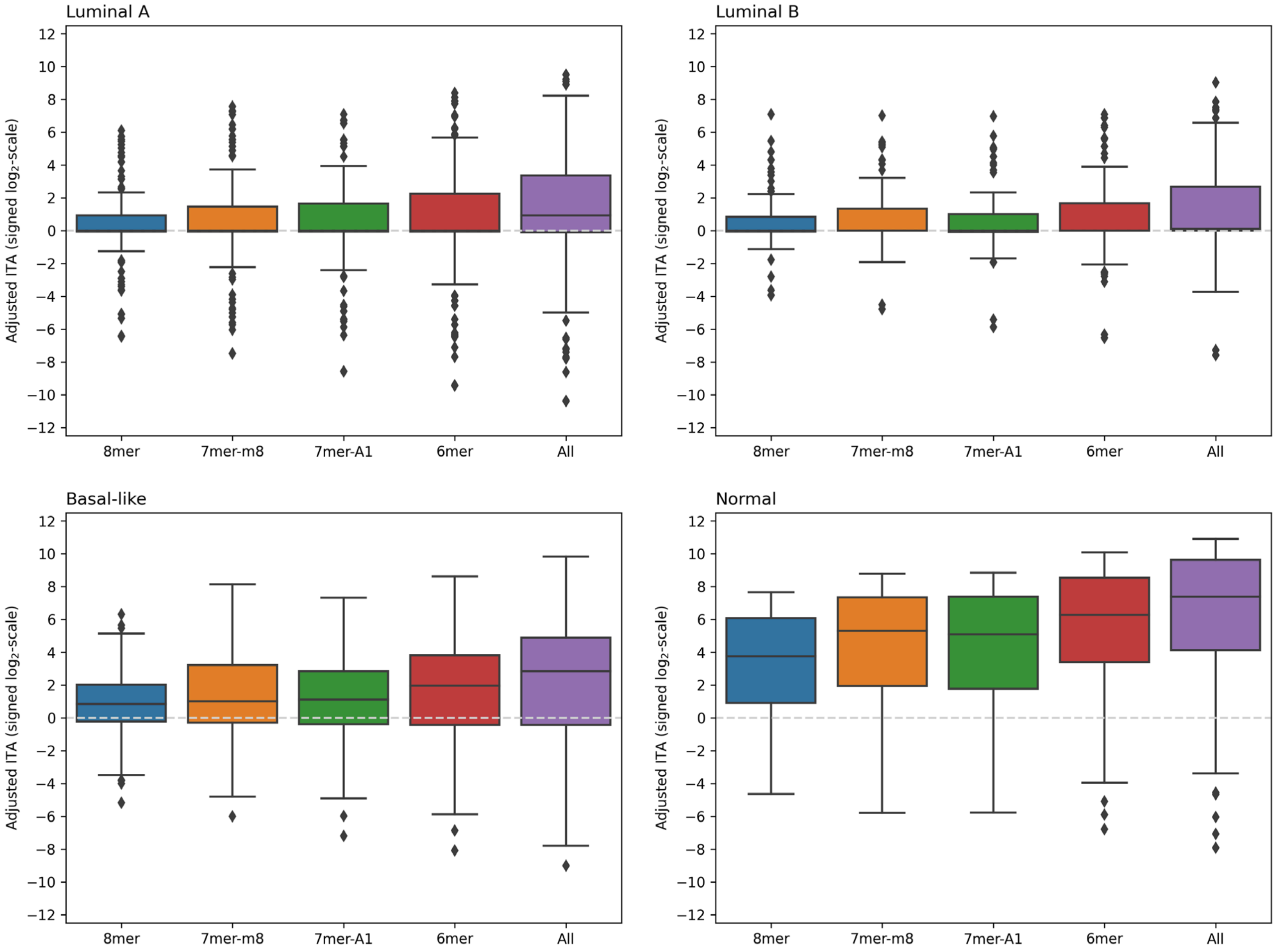

Supplementary Figure 2. The distribution of adjusted ITA values in four groups of samples (only TargetScan predictions). Adjusted ITA values (y-axis) were calculated for each 5′-isomiR and reflect the number of anti-correlated predicted targets adjusted for the background anti-correlations (background was estimated using non-target transcript sequences). To embed positive and negative adjusted ITA values in the logarithmic scale, we applied the following signed log transformation: $sgn(y) * log_2(|y| + 1)$.

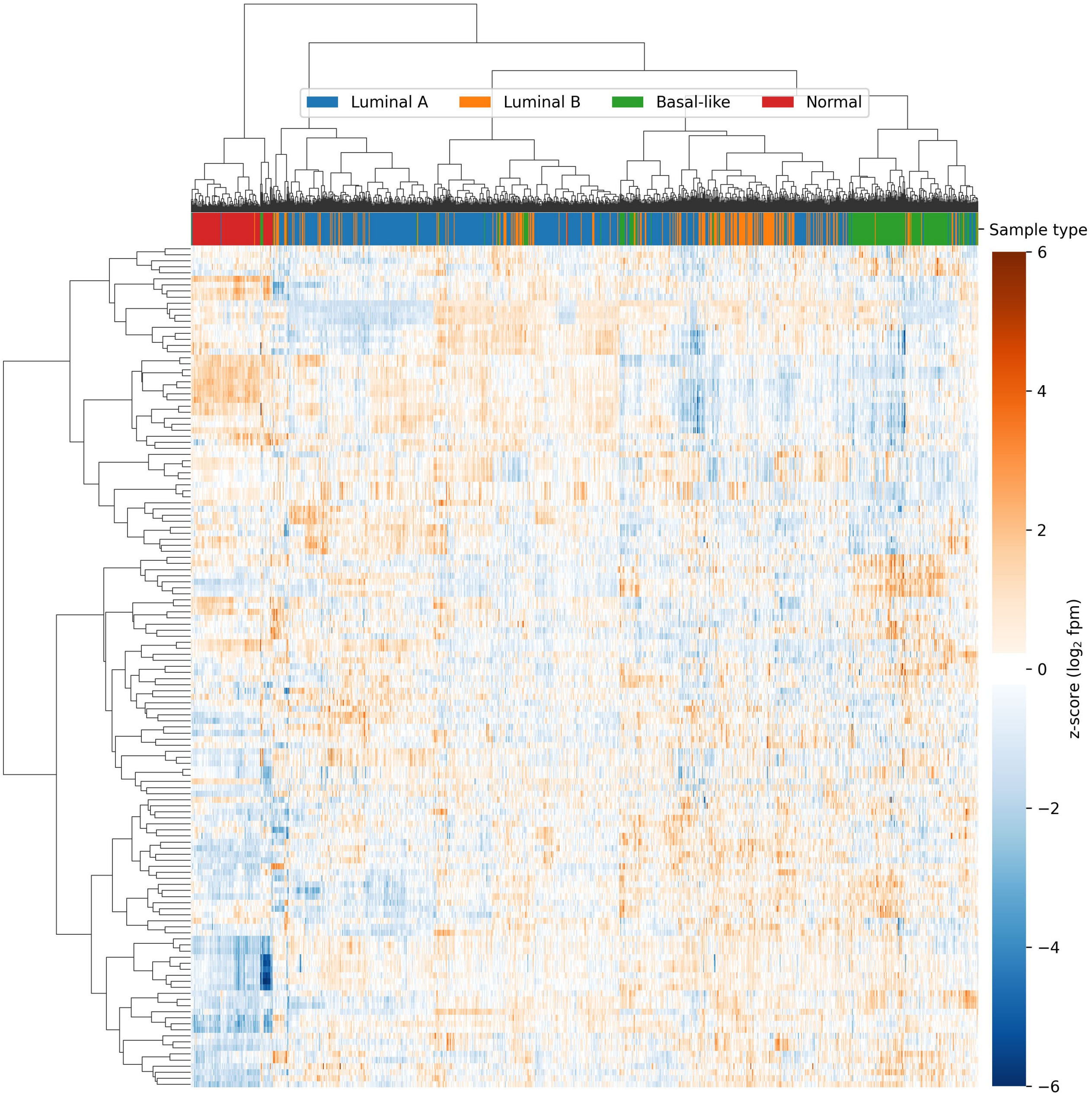

Supplementary Figure 3. Hierarchical clustering of samples (horizontal axis) and 5′-isomiRs (vertical axis). Prior to clustering expression levels of 139 considered 5′-isomiRs were standardized row-wise. Ward's method was used for the clustering and construction of dendrograms.

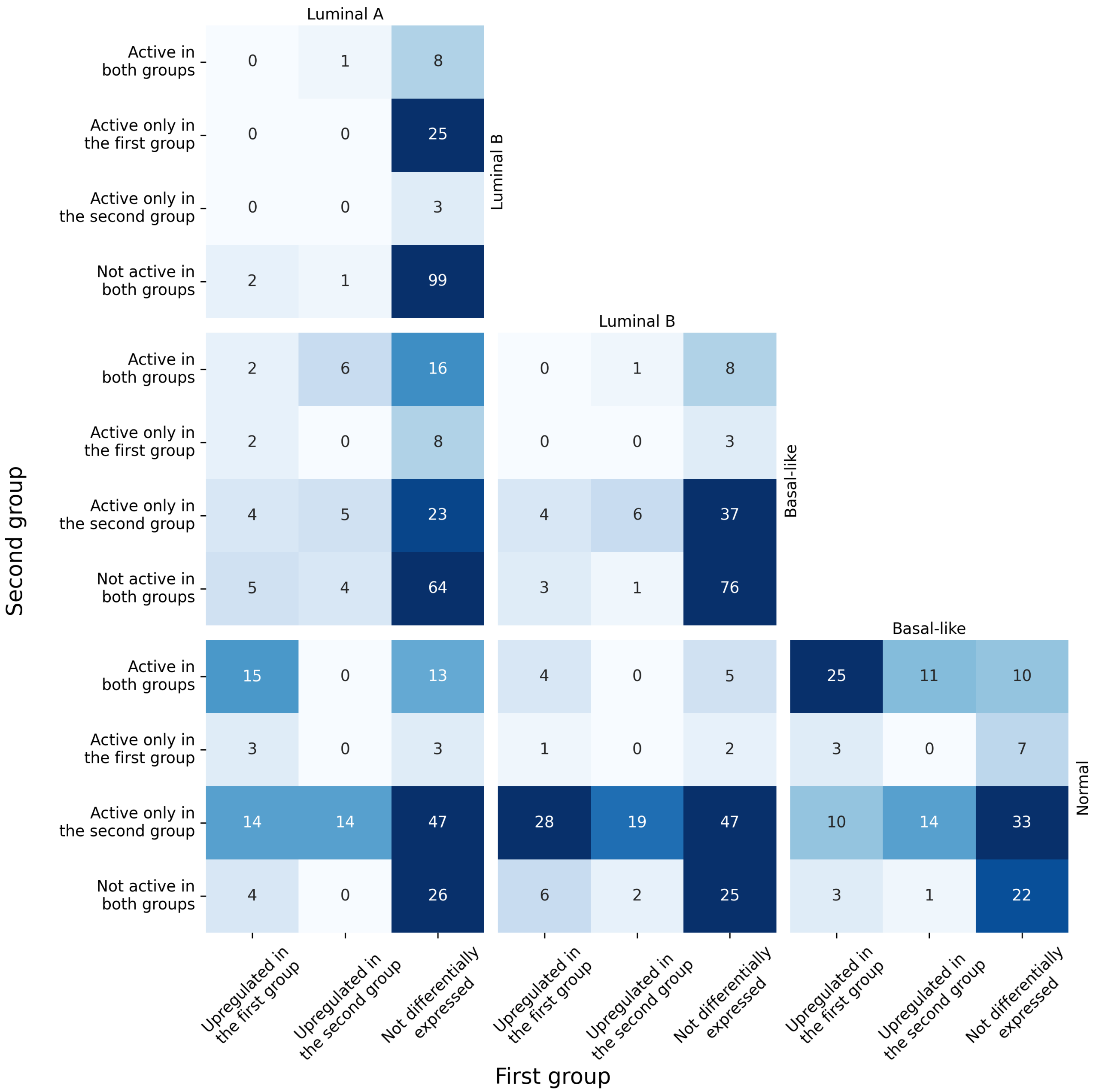

Supplementary Figure 4. Differential 5′-isomiRs activity and expression in four analyzed sample groups. Vertical axis stands for the activity analysis ("active" means adjusted ITA FDR < 0.05). Horizontal axis stand for the differential expression analysis. Sum of all values in each 4 x 3 block (i.e., each pair of sample groups) is equal to 139 – the total number of analyzed 5′-isomiRs.
